# Methods for sample size determination in cluster randomized trials

**DOI:** 10.1093/ije/dyv113

**Published:** 2015-07-11

**Authors:** Clare Rutterford, Andrew Copas, Sandra Eldridge

**Affiliations:** ^1^Centre for Primary Care and Public Health, Barts and the London School of Medicine and Dentistry, Queen Mary University of London, London, UK and; ^2^Hub for Trials Methodology Research, MRC Clinical Trials Unit at University College London, London, UK

**Keywords:** Sample size, cluster randomization, design effect

## Abstract

**Background:** The use of cluster randomized trials (CRTs) is increasing, along with the variety in their design and analysis. The simplest approach for their sample size calculation is to calculate the sample size assuming individual randomization and inflate this by a design effect to account for randomization by cluster. The assumptions of a simple design effect may not always be met; alternative or more complicated approaches are required.

**Methods:** We summarise a wide range of sample size methods available for cluster randomized trials. For those familiar with sample size calculations for individually randomized trials but with less experience in the clustered case, this manuscript provides formulae for a wide range of scenarios with associated explanation and recommendations. For those with more experience, comprehensive summaries are provided that allow quick identification of methods for a given design, outcome and analysis method.

**Results:** We present first those methods applicable to the simplest two-arm, parallel group, completely randomized design followed by methods that incorporate deviations from this design such as: variability in cluster sizes; attrition; non-compliance; or the inclusion of baseline covariates or repeated measures. The paper concludes with methods for alternative designs.

**Conclusions:** There is a large amount of methodology available for sample size calculations in CRTs. This paper gives the most comprehensive description of published methodology for sample size calculation and provides an important resource for those designing these trials.

Key Messages

- There is a large body of literature on sample size calculations for cluster randomized trials.
- There are relatively simple and accessible methods to allow for design complexities such as variable cluster sizes; time-to-event outcomes; incorporation of baseline values and cross-over, stepped-wedge and matched designs.
- This is the most comprehensive resource to date for sample size methods for cluster randomized trials.
- There is scope for further methodological development.

## Introduction

### 

#### Cluster randomized trials

In a cluster randomized trial, groups or clusters, rather than individuals, are randomly allocated to intervention groups. This approach may be deemed necessary; if randomization at individual level is impractical, to avoid contamination between treatment groups, i.e. individuals in the control arm being exposed to the intervention; or for administrative or cost advantages. The rationale for cluster randomized trials has been described in detail elsewhere.^1–10^

The responses from individuals within a cluster are likely to be more similar than those from different clusters. This is because individuals within a cluster may share similar characteristics or be exposed to the same external factors associated with membership to a particular cluster. This lack of independence introduces complexity to the design and analysis. The degree of similarity, or clustering, is commonly quantified by the intracluster correlation coefficient (ICC) denoted in this article as ρ.

Obtaining a good sample size estimate is particularly important in cluster randomized trials due to the large cost that can be associated with recruiting an additional cluster as compared with recruiting an additional subject in an individually randomized trial. Equally important are the ethical implications of over- or under-recruitment where the addition or loss of one cluster may equate to a large number of individuals potentially being exposed to the risk of treatment, or lost.

#### A simple approach to sample size calculation

A consequence of clustering is that the information gained is less than that in an individually randomized trial of the same size, making randomization by cluster less efficient. This inefficiency was identified in the seminal paper by Cornfield that sparked the development of methodology for the design and analysis of cluster randomized trials.^11^ It has been proposed by Donner, Birkett and Buck that a sample size calculated assuming individual randomization can be inflated by a Design Effect (DE) to reach the required level of statistical power under cluster randomization:^12^

(1) DE=1+(n−1)ρ

where n is the number of individuals per cluster and ρ the ICC.

Therefore for a comparison of means, in a two-arm trial with equal allocation the required the number of individuals per group, m, is calculated as:

(2) $$\text{m}=\frac{{\left({\text{Z}}_{1-\alpha /2}+{\text{Z}}_{1-\beta }\right)}^{2\;}2\sigma^{2}}{\Delta^{2}}\left(1+(n-1)\rho \right)$$

where ${\text{Z}}_{\text{x}}$ is the *x*’th percentage point of the standard normal distribution, Δ the clinically important difference in treatment means and $\sigma^{2}$ the variance in the outcome.

Analyses may be conducted at either the cluster or individual level (see Eldridge and Kerry for a full discussion of analysis methods^1^)

In cluster-level analyses, a cluster-level summary is calculated for each cluster, effectively reducing the data to one observation per cluster. The observations can then be treated as independent, and standard statistical analysis methods applied. The main advantages of cluster-level analyses are their simplicity and applicability to different types of outcomes. Disadvantages of this approach are that individual-level covariates cannot be included and the number of observations per group may be small. However, the two-sample t-test has been shown to be quite robust to deviations from normality and a small number of clusters per treatment group.^13^

Methods that use individual-level data but adjust for clustering can be used for analysis, such as the adjusted chi-square method for binary data, the adjusted two-sample t-test^2^ or the non-parametric clustered Wilcoxon test for continuous data.^14^ In this article, these are referred to as adjusted tests. The main drawback to these methods is that they do not allow for the inclusion of covariates.

Commonly individual-level analyses are conducted using a regression model that accounts for the clustered nature of the data and may include either cluster or individual level covariates. Mixed effects regression models are a cluster-specific method (henceforth referred to as mixed models) and Generalised Estimating Equations (GEE), a type of population-averaged or marginal method. Both approaches require a sufficient number of clusters for optimal performance; when the number of clusters is small, the mixed model is less biased than the GEE. The difference between these two approaches lies in the interpretation of the estimated treatment effect.^1^

In general, sample size requirements depend upon the proposed analysis method. In this paper we describe each sample size method alongside the analysis method for which it was designed. However, alternative analysis approaches may also be suitable. For example, with continuous outcomes a cluster-level analysis is equivalent to an individual-level analysis if all the clusters are the same size. When cluster size is variable, the assumptions underlying the cluster-level t-test are not met and a weighted t-test must be used to achieve adequate power and precision. Individual-level analyses naturally incorporate this weighting and so are more efficient than cluster-level analyses weighted by cluster size.^4^ For continuous outcomes and equal-sized clusters, the cluster-specific and population-averaged methods for individual-level analyses are mathematically equivalent.

For binary outcomes, due to the transformation of the data onto the logistic scale, the treatment effects calculated under the cluster-specific and population-averaged methods are different. For binary outcomes, Austin *et al.*^15^ compared the performance of three cluster-level methods: the t-test, the Wilcoxon rank-sum test and the permutation test, and three individual-level methods: the adjusted chi-square test, the mixed effects model and the GEE model. In the scenarios investigated, which included variable cluster sizes, the difference in power between these methods was negligible.

#### Measuring variability between clusters

A key parameter common to all sample size calculations for cluster randomized trials is the extent of similarity between units within a cluster. The measure used in the majority of sample size methodology is the ICC, usually denoted by the Greek letter ρ. The ICC can be interpreted as the proportion of variance due to between-cluster variation. When ρ=0 there is statistical independence between members of a cluster, whereas when ρ=1, all observations within a cluster are identical. A review of estimators for calculating the ICC for continuous and dichotomous outcomes can be found in the papers by Donner^16^ and Ridout,^17^ respectively. Properties of the ICC have been widely investigated and patterns in ICCs^18–22^ and sources of ICC estimates^5^^,^^23–26^ are available in the literature and have been summarized by Eldridge and Kerry.^1^ An alternative measure to the ICC is the coefficient of variation in the outcome, denoted by k. This is calculated as the between-cluster standard deviation divided by the parameter of interest, i.e. the proportion, rate or mean, within each cluster.^27^ This measure is particularly useful when the primary outcome variable is a rate, as an ICC cannot be calculated.^27^

When choosing an estimate of the ICC, in addition to the method of calculation, it is also important to identify whether the estimate has been adjusted for covariates. This can impact on its value and hence on the calculated sample size. Inclusion of the baseline value of an outcome as a covariate is arguably the strongest factor to reduce the ICC. However, this level of detail is not always explicitly reported alongside the ICC estimate.

#### Comparison of ICC and coefficient of variation

Sample size calculations often make the assumption that the measure of correlation, be it the ICC or k, is the same in each treatment group. However, if the coefficient of variation is the same in each treatment group the ICC will not be, and vice versa.^4^ Therefore the use of these different measures will produce different sample size requirements. The assumption of a constant ICC is reasonable if the intervention effect is likely to be constant across clusters. The assumption of a constant k is reasonable if the intervention effect is likely to be proportional to the cluster mean.^1^

Similarly for binary outcomes, different sample size requirements are calculated depending upon whether the ICC or coefficient of variation is used in the calculation. For binary outcomes there is an additional complication that the between-cluster variance also depends upon the value of the overall outcome proportion. The use of the ICC is recommended for sample size calculations of binary outcomes, unless the proportion is very small.^1^

#### Trial design features that impact on sample size

The most common and simplest design choice for a cluster randomized trial is the completely randomized, two-arm parallel-group design with fixed cluster sizes. In this paper, the methods appropriate for this design are discussed first. Variations to this design may be somewhat outside the investigator’s control, such as variability in cluster size or attrition, or more within the investigator’s control, such as choice of outcome measure or analysis method. With these variations, the assumptions of constant cluster size, binary or continuous outcomes, and ICC underpinning the use of the simple design effect,(1) may not be met; appropriate approaches are presented. The paper concludes with the presentation of methods for alternative design choices such as the cross-over, stepped-wedge, matched and three-level designs.

Sample size methodology covering some of these aspects has been summarized^1–5^^,^^27^ and Campbell *et al.* have discussed some of the complexities including: methods for survival data; allowing for imprecision in the estimate of the ICC; allowing for varying cluster sizes; sample size re-estimation; empirical investigations of design effect values; and adjusting for covariates.^28^ However, currently there is no single resource for researchers designing cluster randomized trials that provides a comprehensive description of existing published sample size methodology. Our work is based on an assessment of the literature. A description of how the papers were identified and included can be found in our online appendix (available as Supplementary data at *IJE* online). This article aims to provide both a summary of methods and practical guidance around the use of different methods.

## Results: sample size methods

Where possible, sample size formulae have been re-expressed to use consistent terminology for ease in comparability. Due to limited space within this manuscript, if implementing some of the more complex methods or those whose components require detailed description, readers are advised to refer to original papers for further information and to ensure correct implementation and understanding of the methodology.

Sample size methods are now presented, starting with the standard parallel-group trial, followed by variations to this design and concluding with alternative designs.

### Standard parallel-group, two-arm design

#### Continuous and binary outcomes

Table 1 summarizes the methodology available for the standard parallel-group trial with equal sized clusters.

**Table 1.** Sample size methods for the standard two-arm, parallel group, equal allocation, fixed cluster sizes completely randomized design

Standard trial design	Outcome measure	Analysis	Reference
Two-arm, parallel-group, completely randomized design	Continuous	Cluster-level	^12^^,^^27^^,^^30–32^
		Adjusted test	^33^
		Mixed model	^76^
		GEE	^29^
	Binary	Cluster-level	^11^^,^^12^^,^^27^^,^^30–32^
		Mixed model	^78^
		GEE	^29^
	Count	GEE	^34^
	Ordinal	GEE	^35^
		Mixed model	^36^
	Time-to-event	Cluster-level	^39^^, ^^103^
		Mixed model	^40^
		Marginal model	^43^
		Marginal model	^42^
	Rate	Cluster-level	^27^

The standard design effect or equivalent has been developed for continuous and binary outcomes, analysed at the cluster-level, or at individual level using a GEE model.

For continuous outcomes, the number of individuals per arm, m, is calculated as^12^^,^^29^

(3) $$\text{m}=\frac{{\left({\text{Z}}_{1-\alpha /2}+{\text{Z}}_{1-\beta }\right)}^{2\;}2\sigma^{2}}{\Delta^{2}}\;[1+\left(n-1\right)\rho ]$$

where ${\text{Z}}_{\text{x}}$ is the x’th percentage point of the standard normal distribution, Δ represents the clinically important difference in treatment means,$\;\sigma^{2}\;$the total variance in the outcome, n the cluster size and ρ the ICC.

Alternatively, the number of clusters per arm, c, for a cluster-level analysis can be estimated using direct estimates of the between- and within-cluster variances, $\sigma_{b}^{2}$ and $\sigma_{w}^{2}$.^30–32^

(4) $$\text{c}=\frac{{\left({\text{Z}}_{1-\alpha /2}+{\text{Z}}_{1-\beta }\right)}^{2\;}2(\sigma_{b}^{2}+\frac{\sigma_{w}^{2}}{n})}{\Delta^{2}}$$

Rosner and Glynn^33^ present sample size methods for non-normally distributed continuous outcomes analysed with an adjusted test, the clustered Wilcoxon test. This method requires a large number of calculations but can be implemented using SAS macros provided by the authors.

For binary outcomes, the number of individuals per arm, assuming a cluster-level analysis, is calculated as^12^

(5) $$\begin{array}{l} \text{m}=\frac{{\left({\text{Z}}_{1-\alpha /2}+{\text{Z}}_{1-\beta }\right)}^{2\;}[{\text{P}}_{1}\left(1-{\text{P}}_{1}\right)+{\text{P}}_{2}(1-{\text{P}}_{2})]}{\Delta^{2}}\;\\ \times [1+\left(n-1\right)\rho ] \end{array}$$

where ${\text{P}}_{1}$ is the probability of an event in the control group, and ${\text{P}}_{2}$ the probability of an event in the treatment group, and Δ represents the clinically important difference in treatment proportions, ${\text{P}}_{1}-{\text{P}}_{2}$. The design effect can also be used to inflate the variance for the treatment effect described by a log odds ratio and assuming a GEE analysis.^29^

Alternatively, the number of clusters per group, assuming a cluster-level analysis can be calculated as^30^^,^^31^

(6) $$\text{c}=\frac{{\left({\text{Z}}_{1-\alpha /2}+{\text{Z}}_{1-\beta }\right)}^{2\;}[2\sigma_{b}^{2}+\frac{{\text{P}}_{1}\left(1-{\text{P}}_{1}\right)+{\text{P}}_{2}(1-{\text{P}}_{2})}{n}]}{\Delta^{2}}$$

Simple methods are available for continuous and binary outcomes that use the coefficient of variation in outcome as a measure of correlation and assume a cluster-level analysis.^27^ For continuous outcomes where $\mu_{1}$ and $\mu_{2}$ are the means in the control and intervention group, respectively, and $\sigma_{1}$ and $\sigma_{2}$ the associated within-cluster standard deviations, the number of clusters per group is shown as

(7) $$c=1+\frac{{\left({\text{Z}}_{1-\alpha /2}+{\text{Z}}_{1-\beta }\right)}^{2}\left[\frac{\left(\sigma_{1}^{2}+\sigma_{2}^{2}\right)}{n}+\;k^{2}\left(\mu_{1}^{2}+\mu_{2}^{2}\right)\right]}{{\left(\mu_{1}-\mu_{2}\right)}^{2}}$$

Similarly for binary outcomes where ${\text{P}}_{1}$ and ${\text{P}}_{2}$ are the proportions in the control and intervention group, respectively,

(8) $$c=1+\frac{{\left({\text{Z}}_{1-\alpha /2}+{\text{Z}}_{1-\beta }\right)}^{2}\left[\frac{P_{1}(1-P_{1})}{n}+\frac{P_{2}(1-P_{2})}{n}+k^{2}\left(P_{1}^{2}+P_{2}^{2}\right)\right]}{{\left(P_{1}-P_{2}\right)}^{2}}$$

One cluster per group has been added to account for the use of the normal approximation in the sample size calculation.

#### Count outcomes

For count outcomes, multiplication of the sample size calculation for ordinary Poisson regression by the standard design effect can be used to calculate the number of individuals per group, m, assuming fixed cluster size, and an analysis by GEE^34^

(9) $$m=\frac{{\left[{\text{Z}}_{\alpha /2}\sqrt{2}+{\text{Z}}_{\beta }\sqrt{\left[1+e^{-\tilde{b}}\right]}\right]}^{2}}{e^{\beta_{0}}{\tilde{b}}^{2}}[1+\left(n-1\right)\rho ]$$

where $\beta_{0}$ represents the event rate in the control group and $\tilde{b}$ is the treatment effect.

#### Ordinal outcomes

A method for correlated ordinal outcomes assuming a GEE analysis has been proposed.^35^ This method has been described in the context of longitudinal data where the number of repeated measurements (or cluster size) is small and the number of clusters large. Its performance for smaller numbers of larger clusters is unknown and its implementation is best done via computer. More recently, Campbell and Walters^36^ suggest multiplication of Whitehead’s sample size calculation for ordinal outcomes in individually randomized trials by the design effect^37^

(10) $$m=\frac{6{\left[z_{1-\alpha /2}+z_{1-\beta }\right]}^{2}/{\left(\log OR\right)}^{2}}{\left[1-\overset{I}{\underset{i=1}{\sum }}{\bar{\pi }}_{i}{}^{3}\right]}\;[1+(n-1)\rho ]$$

${\bar{\pi }}_{i}$ is the mean proportion expected in ordinal category i calculated as ${\bar{\pi }}_{i}=(\pi_{1i}+\pi_{2i})/2$ where $\pi_{1i}$ and $\pi_{2i}$ are the proportions in category i for the control and intervention groups. The treatment effect is given by the log odds ratio and a mixed model analysis is assumed.

#### Time-to-event outcomes

Methods have been suggested for time-to-event outcomes that adapt the formulae for individual randomization provided by Schoenfeld.^38^

The required number of individuals per group given by Schoenfeld’s formula for individually randomized trials assuming equal allocation is

(11) $${\text{m}}_{0}=\frac{2{\left({\text{Z}}_{1-\alpha /2}+{\text{Z}}_{1-\beta }\right)}^{2}}{{\text{log}}_{e}^{2}\theta \;(1-P\left(C\right))}$$

where P(C) is the probability of being censored and θ denotes the hazard ratio.

The standard design effect can be used to inflate the formula of Schoenfeld assuming the cluster-level weighted log-rank test.^39^

Jahn-Eimermacher *et al.*^40^ present a simple formula for time-to-event outcomes adjusting Schoenfeld’s formula and using the coefficient of variation in outcome as a measure of clustering and assuming a mixed model analysis using a shared frailty model, a popular method for the analysis of clustered time-to-event data. The number of clusters per group is given by

(12) $$C\approx m_{0}+{\left(Z_{\alpha /2}+Z_{\beta }\right)}^{2}k^{2}\frac{1+\theta^{2}}{{\left(1-\theta \right)}^{2}}$$

where $m_{0}$ is the required number of clusters per group assuming uncorrelated data according to Schoenfeld (11) and k is the coefficient of variation in outcome.

Alternatively, Freedman’s formula^41^ for the number of events required under individual randomization can be multiplied by the design effect^42^

(13) $$E={\left({\text{Z}}_{1-\alpha /2}+{\text{Z}}_{1-\beta }\right)}^{2}\frac{{\left(1+\theta \right)}^{2}}{{\left(1-\theta \right)}^{2}}[1+\left(\bar{n}-1\right)\rho ]$$

where $\bar{n}$ is the average cluster size, and analysis by marginal model is assumed.

Manatunga^43^ considers time-to-event outcomes also assuming a marginal model, although the method does not provide a simple explicit formula.

#### Rate outcomes

The number of clusters per group, c, for rate outcomes in an unmatched design with cluster-level analysis is^27^

(14) $$c=1+\frac{{\left({\text{Z}}_{1-\alpha /2}+{\text{Z}}_{1-\beta }\right)}^{2}\left[\frac{r_{1}+r_{2}}{y}+\;k^{2}\left(r_{1}^{2}+r_{2}^{2}\right)\right]}{{\left(r_{1}-r_{2}\right)}^{2}}$$

where y is the number of person-years in each cluster (assumed equal), k the coefficient of variation in the outcome and ${\text{r}}_{1}$ and ${\text{r}}_{2}$ the rates in the control and intervention group, respectively.

### Variations to the standard parallel-group design

Table 2 provides a summary of all sample size methodology for variations to the standard parallel group trial. The key methods in each area are presented and discussed here.

**Table 2.** Sample size methodology for adaptations to the standard two-arm, parallel-group, completely randomized design

Adaptation	Outcome measure	Analysis	Reference
**Design**			
ICC uncertainty	Continuous	Cluster-level	^45^
		Adjusted test	^49^
		Mixed model	^44–46^
		GEE	^45^^,^^46^
	Binary	Cluster-level	^47^
Variable cluster sizes	Continuous	Cluster-level	^50^^,^^51^^,^^61^
		Adjusted test	^55^
		Mixed model	^56^
		GEE	^53^
	Binary	Cluster-level	^50^^,^^51^^,^^105^
		Adjusted test	^54^
		Mixed model	^57^
		GEE	^52^^,^^53^
	Time-to-event	Cluster-level	^103^
Internal pilot	Continuous	Mixed-model	^58^
		GEE	^59^
	Binary	GEE	^59^
Unequal allocation ratio	Continuous	Cluster-level	^61^
		Mixed model	^60^
Small number of clusters	Continuous	Cluster-level	^13^^,^^107^
	Binary	Cluster-level	^13^
Equivalence	Continuous	Adjusted test	^36^
	Binary	Adjusted test	^63^
Non-inferiority	Binary	Adjusted test	^64^
**Conduct**			
Attrition	Continuous	Adjusted test	^65^
		Mixed model	^66^
	Binary	Adjusted test	^65^
Non-compliance	Binary	Adjusted test	^64^^, ^^67^
**Analysis**			
Inclusion of covariates	Continuous	Cluster-level	^70^^,^^71^
		Mixed model	^69^^,^^74–76^^,^^79^^,^^81^^,^^108^
		GEE	^53^^,^^73^^,^^108^
	Binary	Mixed model	^69^^,^^74^^,^^80^
		GEE	^53^^,^^72^^,^^73^^,^^104^ ^108^
Inclusion of repeated measures	Continuous	Mixed model	^66^^,^^82–84^^,^^86^
		GEE	^85^
	Binary	GEE	^85^

#### Uncertainty around the estimate of the ICC

There is often large uncertainty around the estimate of the ICC, leading to wide confidence intervals. As the value of the ICC has a large impact upon the required sample size, it is sensible to consider the impact of its uncertainty. An informal method to address this problem has been to use a conservative estimate of the ICC in the sample size calculation; this provides a quick gauge of the impact of the ICC but could lead to unnecessarily large trials. Several authors have proposed formal methods of incorporating ICC uncertainty into the sample size calculation by making distributional assumptions for one or many previously observed ICC values and then calculating the corresponding distribution for the power.^44–47^ Several of these methods adopt a Bayesian perspective but assume the analysis will follow a frequentist approach. Incorporating uncertainty about the ICC into the sample size calculation produces larger sample sizes than using a single estimate.

There may be situations where there are no good estimates of the ICC available for sample size calculations. This occurred in a trial of mental illness because the outcome measure was a newly adaptive questionnaire with unknown properties.^48^ In these situations, several approaches might be considered: an educated estimate could be gained from assessment of published ICCs and known patterns in their behaviour for different outcome types and clusters; graphical methods that compare competing designs without requiring knowledge of the ICC^49^; or an internal pilot could be considered (see later section).

#### Variable cluster sizes

The use of the standard design effect assumes that the number of observations from each cluster to be included in the analysis is the same. In some situations such as ophthalmology studies where the cluster is a person and measurements are taken on eyes, this may be a reasonable assumption. However, in trials of primary care where the cluster may be a general practice or drop out may occur within clusters, it is more likely that clusters of variable size will be present in the analysis, and it is good practice to consider the potential impact of this at the design stage. If cluster sizes are variable, the use of the mean cluster size in the simple design effect will underestimate the required sample size, more so as the variation in cluster sizes increases. Use of the maximum cluster size as an alternative may be overly conservative. Methods to account for variable cluster size are recommended when cluster size variability is large, i.e. the coefficient of variation of cluster size, defined as the ratio of the standard deviation of cluster size ${\text{S}}_{\text{n}}$ to mean cluster size $\bar{n}$, is greater than 0.23.^50^

The available methods to account for variable cluster size can be divided into two groups: I, those that require the size of each cluster to be known and II, those that require the mean and standard deviation of the distribution of cluster size.

#### Methods that require the size of each cluster to be known:

Here the design effect is given by

(15) $$DE=\frac{\bar{n}c}{\overset{c}{\underset{i=1}{\sum }}\frac{n_{i}}{1+(n_{i}-1)\rho }}$$

where c represents the number of clusters per group, ${\text{n}}_{\text{i}}$ the size of cluster i and $\bar{n}$ mean cluster size.

This DE is appropriate for a cluster-level analysis with minimum variance weighting for continuous or binary outcomes.^51^ It is also applicable for an analysis by GEE with exchangeable correlation structure, robust variance estimators and binary outcomes.^52^ By exchangeable correlation we mean that every subject within a cluster is equally correlated to every other subject and this pair-wise correlation is denoted ρ. This is a common and reasonable assumption to make for cluster randomized trials. An alternative approach is to assume that the within-cluster correlation can be specified by an identity matrix, also known as the working independence model. This correlation offers advantages, in that for model fitting it is simple and can aid model convergence. If the working independence model was assumed but the true correlation was exchangeable, then the following design effect can account for this misspecification^52^

(16) $$DE=\frac{\bar{n}c\overset{c}{\underset{i=1}{\sum }}n_{i}\left(1+(n_{i}-1)\rho \right)}{{\left(\overset{c}{\underset{i=1}{\sum }}n_{i}\right)}^{2}}$$

In the case of equal cluster sizes, this method reduces to the standard design effect and the use of the working independence model results in no loss in efficiency. These GEE methods may be less appropriate for small samples, as the robust variance estimator does not perform well in this situation. Pan^52^ recommends that potential misspecification of the correlation structure be explored at the design stage; please refer to the paper for further examples of alternative combinations of working and true correlation structures.

A sample size method that can accommodate variable cluster sizes and allow adjustments for covariates analysed with a GEE model has been proposed by Liu.^53^ However, except in some special cases (equal cluster sizes and only treatment fitted in the model), there is no closed form available and the method must be implemented numerically. For an exchangeable correlation structure with fixed cluster size, the methods of Liu and Pan can be compared; Pan's method has been shown to produce marginally larger sample sizes.^52^ The difference comes from the use of the score test by Liu compared with the Wald test in the derivation by Pan.

#### Methods that require only the mean and standard deviation of the distribution of cluster size:

It is not common to have knowledge about each cluster size at the design stage. Estimates of the distribution (mean and standard deviation) of cluster size are likely to be more available. However, it should be noted that, in some cases, the mean and SD of the sampling distribution may be different from those of the population distribution of all clusters. The design effect is now

(17) $$DE=1+\left\{\left(CV^{2}+1\right)\bar{n}-1\right\}\rho$$

CV is the coefficient of variation of cluster size.

This design effect can be used with an appropriately weighted cluster-level analysis for binary or continuous outcomes.^50^^,^^54^^,^^55^As individual-level analyses are more efficient, it provides an overestimate of sample size required for most individual level analyses.

Van Breukelen^56^ and Candel^57^ propose the total number of clusters, as computed assuming equal cluster size and mixed model analysis, multiplied by the following design effect to account for variability in cluster size. It potentially has wide applicability as the authors suggest its use for correction of sample sizes calculated using any current formulae where equal-sized clusters are assumed.

(18) $$DE\approx \frac{1}{1-CV^{2}\frac{\bar{n}}{\bar{n}+\frac{1-\rho }{\rho }}\left[1-\frac{\bar{n}}{\bar{n}+\frac{1-\rho }{\rho }}\right]}$$

The above DE is calculated via Taylor approximation but is considered to provide a good approximation for all reasonable distributions of cluster size. Heterogeneous variances across treatment groups can also be accommodated.^57^

#### Internal pilots

For trials that recruit a relatively large number of clusters over a fairly long period of time, it may be appropriate to re-estimate the sample size during the trial once information has been gained on the ICC and other nuisance parameters.^58^^,^^59^ These methods assume a mixed model analysis for continuous outcomes and GEE for binary or continuous outcomes. The use of these internal pilots is less common in clustered trials and further investigation is required to determine best practice for their use, for example it is not known at which stage an interim estimate of the ICC can be considered stable and used to adequately re-estimate the sample size.

#### Allocation ratio

Design efficiency is maximized with equal allocation to treatment groups, and this has been assumed in the majority of the methodology presented here. However, there is an argument that unequal allocation may occasionally be desirable, particularly in cases where the costs associated with the intervention are high. Liu studies the optimal allocation of units to treatment group when the cost per cluster varies across the treatment groups, assuming a mixed model analysis.^60^ The optimal cluster allocation ratio depends upon the cost ratio between the treatment and control.

#### Small number of clusters

The majority of the methods assume that a relatively large number of clusters is to be recruited, making the approximation to the normal distribution in the formulae appropriate. When the number of clusters is small, calculations based upon these approximations will likely underestimate the required sample size. In this case the normal distribution can be replaced by the t-distribution or methods based on the non-central t used. Donner^13^ presents a power calculation based upon the non-central t-distribution with a simple non-centrality parameter for cluster-level analyses. Extensions to this non-centrality parameter can additionally allow for unbalanced designs.^61^ As the percentage points of the non-central t-distribution are not routinely available in statistical texts, these methods are best implemented with a statistical package using the code provided by the authors.

Alternatively, Snedecor and Cochran^62^ suggest adding one cluster per arm when testing at the 5% level and the number of clusters is small, which is incorporated into the formulae described by Hayes (equations 7, 8 and 14)^27^ or could be added to the other formulae presented.

In general however, trials with a small number of clusters should be avoided. As well as the difficulties in sample size estimation, many analysis methods do not perform as well with a small number of clusters and imbalance in cluster characteristics across treatment groups is more likely to occur.^1^

#### Equivalence and non-inferiority

Non-inferiority and equivalence designs are less commonly used in cluster randomized trials. The methods presented here assume an analysis using an adjusted test. For equivalence designs, the standard design effect can be applied to the sample size calculated under individual randomization for binary outcomes^63^

(19) $$m=\frac{2P(1-P){\left({\text{Z}}_{1-\alpha }+{\text{Z}}_{1-\beta }\right)}^{2}}{d^{2}}[1+\left(n-1\right)\rho ]$$

where P is the true event proportion in both groups and d represents the equivalence limit for the upper limit of the confidence interval of the difference in intervention proportion, and for continuous outcomes^36^

(20) $$m=\frac{2{\left({\text{Z}}_{1-\alpha }+{\text{Z}}_{1-\beta }\right)}^{2}}{{\left(d/\sigma \right)}^{2}}[1+\left(n-1\right)\rho ]$$

Here we have specified one-sided tests. To be conservative, two-sided tests could be used.

The calculation for the number of clusters per treatment group, c, in a non-inferiority trial with binary outcome, is^64^

(21) $$c=\;\frac{{\left(z_{\alpha }+z_{\beta }\right)}^{2}Var(\log\left(OR\right))}{{\left(\log\left(d\right)-\text{log}(OR)\right)}^{2}}$$

where the relative treatment effect is measured by the odds ratio (OR) of a positive response among compliers and d represents the non-inferiority margin of the OR. This method additionally incorporates non-compliance and, due to this, the variance of this odds ratio is complex to calculate (see original paper).

#### Attrition

In a cluster randomized trial, individuals within a cluster may withdraw from the trial or an entire cluster may withdraw or not recruit any participants. Drop-out of entire clusters is relatively uncommon but could be incorporated into the sample size calculation by the addition of 1 or 2 extra clusters per treatment group.

Attrition among members of a cluster is a more common problem, particularly for cohort samples. Conventional approaches to account for such attrition are to divide the sample size by the anticipated follow-up rate or use the anticipated average cluster size in the calculation. However, these methods overestimate and underestimate, respectively, when cluster follow-up rates are highly variable or the cluster size or ICC is large. A design effect has been proposed for binary or continuous outcomes assuming adjusted tests, i.e. the individual-level t-test or chi-square test suitably adjusted for clustering^65^

(22) DE=[1+(nπ−1)ρ+(1−π)[1+(n−1)τ]ρ]/π

π represents the probability of the outcome being observed. A binary missingness indicator variable is 0 if the outcome is missing and 1 otherwise. τ is the intracluster correlation coefficient for the missingness data mechanism, i.e. at its minimum $\tau =-\frac{1}{n-1}\;$implies that all clusters have identical follow up rates and τ=1 implies all the missingness indicators are the same within a cluster (entire clusters are completely observed or completely missing). Currently estimates for τ are not routinely published with the results of trials and the authors recommend a sensitivity analysis using a range of plausible values.

Roy has also considered attrition for the longitudinal clustered design, assuming analysis with a mixed effects regression model.^66^ The calculation uses an iterative method and allows for a differential drop-out across treatment groups and over time.

#### Non-compliance

Sample size requirements increase as the level of non-compliance increases. Methods which allow for non-compliance, where analysis is by an adjusted test, have been proposed for both non-inferiority and superiority designs.^64^^,^^67^ However, the allowance for non-compliance makes the variance of the treatment effect more complex to calculate. These methods may be less applicable in pragmatic cluster randomized trials where the effect of the intervention is usually assessed in the presence of non-compliance. In a truly pragmatic trial, compliance may not be measured or actively encouraged.^68^

#### Inclusion of baseline measurements

Sample size calculations can be adapted to allow covariates in the analysis, as this may increase power by explaining variability and reducing the between-cluster variation, which is particularly important when the number of available clusters is limited or the cost of recruiting each additional cluster is high. Covariates may be collected at the level of the individual or the cluster and they may be demographic variables, such as age, or baseline measures of the primary outcome. Neuhaus and Segal^69^ suggest, in general, that multiplication of the ICC by the ICC of any individual-level covariate provides an estimate of an adjusted ICC that can be used in the standard design effect, assuming a mixed model analysis.

#### Pre-post design

Inclusion of the baseline measurement of the primary outcome into the analysis is referred to as a pre-post design.

The nature of the correlation in a pre-post design will depend upon the population being sampled, for which there are two types: cross-sectional or cohort sample. With a cross-sectional sample, different individuals are measured at each time point. Here there are two sources of correlation to be accounted for: the correlation of outcomes from individuals within a cluster at the same time point (which can be thought of as the familiar ICC, ρ) and the correlation between baseline and follow-up outcomes for individuals within a cluster (referred to as the cluster auto correlation, $\rho_{c}$). With a cohort sample, the same individuals are measured at baseline and follow-up and the additional correlation across time points on the same individual conditional on the cluster is referred to as the subject autocorrelation,$\;\rho_{s}$.

Assuming a cluster-level ANCOVA, a relatively straightforward design effect can be used for the pre-post design.^70^^,^^71^ The design effect can accommodate either the cross-sectional sample ($\rho_{s}=0$), cohort sample or a mixture of the two^70^

(23) $$\begin{array}{l} DE=[1+\left(n-1\right)\rho ]\\ \times (1-{\left(\frac{n\rho }{1+(n-1)\rho }\rho_{c}+\frac{1-\rho }{1+(n-1)\rho }\rho_{s}\right)}^{2}) \end{array}$$

When the analysis is performed on change from baseline scores the design effect is

(24) $$\begin{array}{l} DE=[1+\left(n-1\right)\rho ]\\ \times 2(1-{\left(\frac{n\rho }{1+(n-1)\rho }\rho_{c}+\frac{1-\rho }{1+(n-1)\rho }\rho_{s}\right)}^{}) \end{array}$$

Preisser^72^^,^^73^ focuses on binary outcomes with a GEE analysis. The number of clusters for the cross-sectional pre-post design is given as

(25) $$\text{c}=\frac{{\left({\text{Z}}_{1-\frac{\alpha }{2}}+{\text{Z}}_{1-\beta }\right)}^{2\;}(\sigma_{1}^{2}+\sigma_{2}^{2})}{n{\left(\left(\pi_{11}-\pi_{10}\right)-(\pi_{21}-\pi_{20})\right)}^{2}}$$

where

$$\begin{array}{l} \sigma_{h}^{2}=[\pi_{h1}\left(1-\pi_{h1}\right)+\pi_{h0}\left(1-\pi_{h0}\right)]\left[1-\left(n-1\right)\rho \right]\\ -2n\rho_{c}\sqrt{\pi_{h1}\left(1-\pi_{h1}\right)+\pi_{h0}\left(1-\pi_{h0}\right)} \end{array}$$

and$\;\pi_{ht}$ is the probability of the outcome for an individual at time t (0 = pre-test, 1 = post-test) from treatment group h (1 = control, 2 = intervention).

In terms of sample size, a cohort sample is more efficient, although it suffers from several drawbacks. To gain noticeable precision, the correlation across time points on the same individual must be fairly substantial. Cohort designs can also suffer from loss to follow-up and therefore require oversampling at baseline and attentive follow-up of individuals.

The sample size efficiency of the cohort design relative to the repeated cross-sectional design with 1 measurement on each individual at each time point, assuming a mixed model, has been quantified as^74^^,^^75^

(26) $$RE=\frac{n\left(1-\rho_{c}\right)\sigma_{b}^{2}+\left(1-\rho_{s}\right)\sigma_{w}^{2}}{n\left(1-\rho_{c}\right)\sigma_{b}^{2}+\sigma_{w}^{2}}$$

#### Inclusion of other covariates

Although the inclusion of covariates can reduce the sample size requirements, there are costs associated with taking additional measurements. In a trial without covariates, suppose the total budget for the trial is summarized via the cost function $T=nCc_{1}+Cc_{2}$, where C is the total number of clusters, n the cluster size, $c_{1}$ the costs per individual and $c_{2}$ the costs per cluster. The number of clusters, C, and the number of individuals, n, which minimize the variance of the treatment estimator, given the budget constraint are given as^76–78^

(27) $$C=\frac{T}{\left(\sigma_{w}/\sigma_{b}\right)\sqrt{c_{1}c_{2}}\;+\;c_{2}}\;,\;n=\frac{\sigma_{w}}{\sigma_{b}}\sqrt{\frac{c_{2}}{c_{1}}}$$

A similar approach can be used with the inclusion of covariates.^76^^,^^79^^,^^80^ Alternatively, power-based calculations are provided by Moerbeek, assuming a mixed model.^81^ The total number of clusters is calculated as

(28) $$\begin{array}{l} N\geq 4\frac{\sigma_{w}^{2}\left(1-\frac{n}{n-1}\rho_{W}^{2}\right)+n\sigma_{b}^{2}\;(1-\rho_{B}^{2}+\frac{1}{n-1}\rho_{W}^{2})}{n}\\ {\times (\frac{z_{1-\alpha /2}+z_{1-\beta }}{\Delta })}^{2} \end{array}$$

where $\rho_{W}^{2}$ and $\rho_{B}^{2}\;$are the within-cluster and between-cluster residual correlations between the outcome and the covariate. $\rho_{W}^{}=0$ for a cluster level covariate.

The additional cost to measure a covariate at the individual level is $c_{1}^{*}$ and the additional cost of measuring a covariate at the cluster level is $c_{2}^{*}$. Therefore the total cost function for individual level covariates becomes

$$T=nC(c_{1}+c_{1}^{*})+Cc_{2}$$

and for cluster level covariates

$$T=nCc_{1}+C(c_{2\;}+c_{2}^{*})$$

The costs associated with and without the covariate can be estimated and compared. The inclusion of covariates is more cost effective when the cost of measurement is small and the correlation between covariates and outcome is large. The formula presented by Moerbeek assumes the covariates are uncorrelated with the treatment condition. When the number of clusters is small, this can be achieved via matching on this covariate, particularly recommended for covariates that vary at the cluster level.^79^

#### Inclusion of repeated measurements

Multiple time points introduce additional components of correlation, as the observations for each cluster will be correlated over time. In a longitudinal cluster randomized trial we have a three-level structure with outcomes measured at specific time points within subjects, within clusters. A three-level mixed effects regression model therefore contains additional fixed effect terms for time and the treatment by time interaction. The sample size methods for these designs are more complex than others and the required estimates may be difficult to find. The hypothesis of interest in these trials is the effect of the intervention over time. Assuming a mixed model, the calculation by Koepsell *et al.*^82^ is based on the non-central-t distribution, with the treatment effect adjusted by a design constant allowing for different hypothesized paths of the intervention effect over time. A formula based upon the Wald test of the interaction term for the number of clusters per arm has been proposed^83^

(29) $$n_{3}=\frac{2\sigma^{2}{\left(z_{1-\alpha /2}+z_{1-\beta }\right)}^{2}(1-\rho_{1})}{n_{2}n_{1}\Delta^{2}\overset{n_{1}}{\underset{k=1}{\sum }}{\left(T_{k}-\bar{T}\right)}^{2}/n_{1}}$$

where $n_{x}$ is the number of units at level x (x=1,2,or 3), T represents the equally spaced time variable and $\rho_{1}$is the correlation among level-one units (see later section on three-level trials for definition).

Roy’s iterative method similarly proposes a test of the treatment by time interaction from a mixed effect model but additionally allows incorporation for a differential drop-out across treatment groups and over time.^66^ Murray proposed that a mixed model with random coefficients is a more appropriate analysis for explicitly modelling more than two time points in the analysis.^84^ The additional random effects make this method more complex than others and, although the authors have provided parameter estimates to aid planning for some outcomes, investigators will likely need to spend time and money sourcing suitable estimates. Sample size formulae for assessing change over time assuming an analysis by GEE have been derived by Liu.^85^ However, except under certain correlation structures, the calculations involved in this method are substantial.

If the effect of treatment is expected to diverge over time, sample size can be calculated for testing the treatment effect at the final time point with incorporation of information from the entire study period assuming a compound symmetry structure and mixed model. This produces smaller sample sizes than an assessment at the final time point only, but the assumptions underpinning this method may limit its widespread application.^86^

### Alternative designs

The above methods are described for the parallel group trial and small variations to this standard design. We now consider methodology for alternative design choices. Table 3 summarizes the available sample size methodology for alternative designs.

**Table 3.** Sample size methodology for alternative designs

Trial design	Outcome measure	Analysis	Reference
Matched/stratified	Continuous	Cluster-level	^27^^,^^32^^,^^109^
		Mixed model	^89^
		Bayesian	^92^
	Binary	Cluster-level	^27^^,^^32^^,^^41^^,^^87^^,^^109^
		Mixed model	^89^
		Adjusted test	^91^
	Rate	Cluster-level	^27^^,^^90^
Cross-over	Continuous	Cluster-level	^93^^,^^106^^,^^107^
		Mixed model	^94^
	Binary	Cluster-level	^106^
	Count	Cluster-level	^106^
Stepped-wedge	Continuous	Mixed model	^95^^,^^96^
Three-level	Continuous	Mixed model	^77^^,^^98^^,^^100^^,^^101^
		GEE	^99^
	Binary	GEE	^99^

#### Stratification and matching

Cluster randomized trials in general recruit a smaller number of units than an individually randomized trial. This can potentially lead to baseline imbalances in cluster characteristics across treatment groups. Matching or stratification can be used to improve similarity in clusters across treatment groups. In a matched-pair design, similar clusters are paired, or matched. One cluster from the pair is allocated to the intervention and the other to the control and a cluster-level analysis conducted. Similarity may be defined on cluster-level characteristics that are thought to affect the outcome, such as size or geographical location. Matching reduces the variance between clusters (within strata or within matched pair) and hence can provide efficiency in sample size. The efficiency gains depend upon the effectiveness of the matching. The sample size for an unmatched cluster randomized trial must be inflated by the following DE in order to have the same precision as the matched study^87^

(30) $$\text{DE}=1/(1-\rho_{\text{x}})$$

Its calculation requires knowledge of the correlation in the outcome between matched pairs, $\rho_{\text{x}}$. This correlation can be estimated from previous studies or from the corresponding correlation for a surrogate variable observed prior to randomization, if any exist, otherwise a range of plausible values can be considered.

In planning a matched trial, it is worth noting that any potential gain in efficiency can be lost if clusters drop out of the study, rendering the matched pair unuseable in the analysis. However, ignoring matching and including all clusters in an unmatched analysis of a matched design has been shown to be valid and efficient in trials that recruit a small number of relatively large clusters.^88^

The required number of cluster pairs, m′, is calculated using the following formula assuming analysis at the cluster level

(31) $$m\prime =\frac{\sigma^{2}{\left(t_{\alpha /2;m\prime -1}+t_{\beta ;m\prime -1}\right)}^{2}}{d^{2}}$$

This is the familiar formula for the paired t-test, where d is the expected difference within pairs, $\sigma^{2}$ the variance of this difference and ${\text{t}}_{\text{x};m\prime -1}$ percentage points of the t distribution with *m*′−1 degrees of freedom.

For continuous outcomes the variance is calculated as

(32) $$2\left(\sigma_{b}^{2}+\;\frac{\sigma_{w}^{2}}{n}\right)$$

where$\;\sigma_{b}^{2}$ is the between-cluster variance within a matched pair and $\sigma_{w}^{2}\;$the within-cluster component of variability.^32^^,^^89^

For binary outcomes the variance is calculated as

(33) $$\frac{P_{1}(1-P_{1})+P_{2}(1-P_{2})}{n}+2\sigma_{b}^{2}$$

where ${\text{P}}_{1}$ the expected proportion in the control arm and ${\text{P}}_{2}\;$the expected proportion in the intervention arm.^41^

The methods by Hayes which use the coefficient of variation in outcome for unmatched trials (equations 7, 8 and 14) can be used for matched trials with two modifications.^27^ Two, rather than one, cluster should be added to account for the use of the normal approximation and k should be replaced with$\;k_{m}$, the coefficient of variation between clusters within the matched pair. The Hayes method for rates can be shown to be equivalent to an earlier approach by Shipley.^90^

Stratification is similar to matching, in that we potentially now have several clusters within each stratum, rather than two as we have in a pair-matched study. This has been addressed for binary outcomes with a straightforward calculation.^91^ For continuous outcomes, Kikuchi and Gittins^92^ follow the less common Bayesian approach to design and analysis. However, as the impact of stratification is difficult to ascertain in advance, recommendations are to ignore it in the sample size calculation, for a more conservative estimate.^1^

#### Cross-over designs

Cross-over designs require a smaller number of clusters than a parallel-group trial and are therefore useful when the availability of clusters is limited. A simple design effect for cluster-level analysis has been presented for the cross-over design in which entire clusters switch treatments during the course of the trial^93^

(34) $$DE=\left(1+\left(\frac{1}{2}n_{1}-1\right)\rho_{2}\right)-\frac{1}{2}n_{1}\eta$$

where $n_{1}$ is the number of participants recruited within each cluster across both time periods$,\rho_{2}$ is the correlation between subjects in the same cluster at the same time point and η is the inter-period correlation. In this design, different subjects from each cluster are included in separate periods of the trial (a cross-sectional sample). The treatment effect is calculated within clusters and therefore between-cluster variance is removed and the design is more efficient than the parallel-group.

Alternatively, each subject could be included in both periods within a cluster (a cohort sample). Here a mixed model is assumed. The treatment effect is calculated within subjects, within clusters, so both between-cluster and between-subject variations are eliminated, making this the most efficient cross-over design with cluster level randomization. The relative efficiency (RE) of the cross-over design with cross-sectional sample over the parallel-group cluster randomized design has been quantified by Rietbergen^94^

(35) $$RE=\frac{\left(1+\left(\frac{1}{2}n_{1}-1\right)\rho_{2}\right)-\frac{1}{2}n_{1}\eta }{1+(n_{1}-1)\rho_{2}}$$

and similarly for the cohort sample

(36) $$RE=\frac{1}{2}\frac{1-\rho_{1}-\rho_{2}}{1+(n_{1}-1)\rho_{2}}$$

where $\rho_{1}$ is the intrasubject correlation.

Although cross-over designs can improve efficiency, the nature of the intervention or condition under study may make them inappropriate, as occurs in individually randomized trials.

#### Stepped-wedge design

The stepped-wedge design is similar to the cross-over design, except that the cross-over of treatments is all in one direction and staggered over time. All clusters receive the control intervention at baseline. At various points during the trial (referred to as steps), one or more clusters will cross over to receive the treatment intervention, with all clusters receiving treatment by the end of the trial. The point at which a cluster, or group of clusters, will cross over is randomly determined at the beginning of the trial.

The main criteria for use of a stepped-wedge design is when the implementation of the intervention can only be performed sequentially across clusters, perhaps due to resource constraints, and when the intervention is believed to do more good than harm and so it would be considered unethical for some clusters to not receive the intervention at some point during the trial. Although these designs are increasing in popularity, there is little published research describing best practice in their design and analysis. Hussey in 2005^95^ provides the first guidance on sample size, which has been further developed by Woertman and assumes analysis by mixed model.^96^

This recently developed sample-size approach for the stepped-wedge design with continuous outcomes supposes that, between each step, one or more cross-sectional sampling waves of the clusters occur and outcome measurements are taken. The total number of individuals required under individual randomization is multiplied by a DE to give the number of individuals to be sampled across all clusters at each sampling wave

(37) $$\begin{array}{l} N_{sw}=\frac{{\left({\text{Z}}_{1-\alpha /2}+{\text{Z}}_{1-\beta }\right)}^{2\;}4\sigma^{2}}{\Delta^{2}}\\ \times [\frac{1+\rho (ktn+bn-1)}{1+\rho \left(\frac{1}{2}ktn+bn-1\right)}\times \frac{3(1-\rho )}{2t\left(k-\frac{1}{k}\right)}] \end{array}$$

where k is the number of steps, b the number of pre-randomization sampling waves, t the number of sampling waves between each step, n the number sampled from each cluster at each sampling wave and ρ is the ICC.$\;N_{sw}$ is the total number of individuals required at each time point, the required number of clusters is calculated as $N_{sw}/n$, the number of clusters switching treatment at each step is calculated by dividing the number of clusters by k and the total number of individuals required across the entire trial is $N_{sw}$ multiplied by (b + kt).

#### Three-level cluster randomized trials

Additional levels of clustering may occur due to the choice of cluster. For example, three-level cluster randomized trials are fairly common in educational research where pupils (level 1 units) are sampled within classrooms (level 2 units) and randomization takes place at the level of the school (level 3 units). The total variance is now made up of the variance between schools, $\sigma_{3}^{2},$ the variance between classrooms within schools, $\sigma_{2}^{2},\;$and the variance associated with students within classrooms and schools, $\sigma_{1}^{2}$ . We can define two ICCs,^97^ for students within schools

(38) $$\rho_{2}=\sigma_{3}^{2}/(\sigma_{3}^{2}+\sigma_{2}^{2}+\sigma_{1}^{2})$$

and for students within classrooms

(39) $$\rho_{1}=\sigma_{3}^{2}+\sigma_{2}^{2}/(\sigma_{3}^{2}+\sigma_{2}^{2}+\sigma_{1}^{2})$$

In a three-level trial, the required sample size is calculated as

(40) $$n_{3}n_{2}n_{1}=DE\times m$$

where m is the number of individuals required in each group in an individual randomized controlled trial (RCT) and $n_{x}$ is the number of units at level x (x=1,2,or 3).

The Design effect for three levels of clustering is

(41) $$DE=1+n_{1}\left(n_{2}-1\right)\rho_{2}+(n_{1}-1)\rho_{1}$$

This DE can be used for continuous outcomes with equal cluster size analysed with either a mixed effects model or GEE assuming exchangeable correlation, as these methods are equivalent under equal cluster size.^98–100^ The design effect in the original paper by Teerenstra^100^ has been re-expressed for the purpose of this paper to use the Pearson correlations (38 and 39), as these are more familiar quantities and published estimates are more likely than the variance components described in the original paper.

Following Raudenbush,^76^ optimization of the sample sizes at each level can be performed based upon cost constraints.^101^^,^^102^

## Discussion

Sample size calculations for individually randomized trials must be inflated in order to be used for cluster randomized trials, to account for the inefficiency introduced by the correlation of outcomes between members of a cluster. A simple design effect described by Donner, Birkett and Buck^12^ can be used for parallel-group trials when the cluster size is assumed constant and the outcome is continuous, binary, count or time-to-event.

Design effects have been derived for more complex designs including: variable cluster sizes; individual level attrition; cross-over trials; stepped-wedge designs; inclusion of baseline measurements; analysis by GEE; and three levels of clustering. These design effects are relatively straight forward to calculate. However, the opportunity to use them may depend upon the availability and quality of estimates of the parameters required for the calculation. When incorporating variable cluster size, the choice of methods depends upon whether every cluster size is known in advance, or just information on cluster size distribution. In the case of incorporating stratification, the only method available requires knowledge about the proportion of individuals in the stratum as well as the success probabilities in each, information which is unlikely to be available at the beginning of the trial. These other parameters, required to assist others planning future trials, are not currently reported as part of a trial’s findings, but we hope will become routinely published in time.

The intracluster correlation coefficient featured more frequently as a measure of within-cluster correlation than the coefficient of variation, in our assessment of the sample size literature. This may be due to the wide availability of published reviews of ICC estimates^5^^,^^23–26^ and patterns in ICCs.^18–22^

The majority of papers specify binary or continuous outcomes; few deal with other types of outcome. Simple approaches for alternative outcomes data potentially warrant future development.

Sample size by simulation is an alternative to using an analytical formula. Although the procedure may be computationally intensive, in some cases it may be preferable to complex numerical procedures and was used in four papers identified in the literature.^103–106^ Many of the methods proposed recommend validation of the sample size calculated with a formula through simulation, particularly for time-to-event outcomes or where the number of clusters is small. However, the type I error is often inflated when the number of clusters is small, the cluster size is variable and for particular analyses such as the frailty model, and this should be taken into consideration during the planning and interpretation of simulations.

We have provided a comprehensive description of sample size methodology for cluster randomized trials, presented in a simple way to aid researchers designing future studies.

With the increasing availability of more advanced methods to incorporate the full complexity that can arise in the design of a cluster randomized trial, the researcher may feel overwhelmed by the volume of methods presented. However it should be noted that in some situations a simple formula may perform reasonably well in comparison with a more complex methodology. For example, when the coefficient of variation in cluster size is less than 0.23, it is not deemed necessary to adjust the sample size and the standard design effect obtained assuming fixed cluster sizes would suffice.^50^

For continuous outcomes with equal cluster sizes, the cluster-level and individual-level analyses are equivalent. Therefore a sample size calculation assuming either of these with the same measure of correlation should produce equivalent results. When cluster size is variable, an individual-level analysis is more efficient than a cluster-level analysis weighted by cluster size; therefore a sample size calculation based upon cluster-level analyses will be somewhat conservative if an individual analysis is then conducted.

For binary outcomes, if the intervention is designed to reduce the outcome proportion use of the coefficient of variation^27^ will produce marginally smaller sample sizes than using the ICC.^12^ When the intervention aims to increase the outcome proportion, the sample sizes using the coefficient of variation will be larger. When several methods may be used, the choice between them is also a question of practicality. The distribution of the outcome and whether required estimates are available should be considered. Further work is required to formally compare the resulting sample sizes calculated under competing methods, when alternative analyses are conducted, and to evaluate the situations in which the simple methods can provide reasonable results over the more complex. This was beyond the scope of this paper.

A limitation of this paper is that a full critique and comparison of the sample size methods were difficult due to the lack of consistency in reporting across the papers. No guidelines exist at present to judge the quality of methodological papers and guide authors in clear and transparent reporting. We hypothesize that the way in which these methods are reported can also be a barrier to their uptake. We hope that their presentation in this article will improve uptake and research in the performance of these methods. We are planning further work looking at developing guidelines for the reporting of methodology papers.

There is often a large amount of uncertainty associated with the estimate of the ICC, and the appropriateness of any of the methods described here will depend upon the level of uncertainty. In the case of a large amount of uncertainty, we recommend that at a minimum the sample size sensitivity to a range of ICC values be explored. We recommend that, at the design stage, an appropriate simple formula be used in the first instance to provide the researcher with a benchmark figure upon which the impact of incorporating further complexities can be assessed.

## Funding

This work was supported by the Medical Research Council.

## Supplementary Material

Supplementary Data
