# Supplementary material for: Methods for sample size determination in cluster randomized trials
Source: Int J Epidemiol. 2015 Jul 11;44(3):1051–67. doi: 10.1093/ije/dyv113 (PMC4521133; doi:10.1093/ije/dyv113)
Supplement: Supplementary Data [file supp_44_3_1051__index.html]

Methods for sample size determination in cluster randomized trials — Methods for sample size determination in cluster randomized trials — Supplementary Data 

# Methods for sample size determination in cluster randomized trials

## Supplementary Data

files

- Supplementary Data - zip file
