# Supplementary material for: Methods for sample size determination in cluster randomized trials
Source: Int J Epidemiol. 2015 Jul 11;44(3):1051–67. doi: 10.1093/ije/dyv113 (PMC4521133; doi:10.1093/ije/dyv113)
Supplement: Supplementary Data [file supp_dyv113_suppl_data.zip › ije-2014-04-0428-File003.docx]

| **Figure 1** Search terms used for the identification of published sample size methodology from the electronic data sources* |
| --- |
| 1. cluster analysis[MeSH] AND sample size[MeSH] 2. ``sample size'' [Title] 3. ``design effect''[Title] OR ``design effects''[Title] OR ``variance inflation factor''[Title] 4. (design*[Title] OR plan*[Title] OR siz*[Title]) AND cluster*[Title] 5. power [title] AND cluster*[Title] 6. ``intraclass correlation*''[Title] OR ``interclass correlation*''[Title] OR ``intracluster correlation*''[Title] OR ``coefficient of variation''[Title] OR ``between cluster''[Title] 7. coefficient[Title] AND variation[Title] 8. (design[Title] OR matching[Title]) AND community[Title] 9. power[Title] AND correlated[Title] 10. number[Title] AND clusters[Title] 11. 1 OR 2 OR 3 OR 4 OR 5 OR 6 OR 7 OR 8 OR 9 OR 10   *note, item 1 was only used in the search of the PubMed database where the indexing by medical Subject Headings (MESH) are available. |
