# Supplementary material for: Methods for sample size determination in cluster randomized trials
Source: Int J Epidemiol. 2015 Jul 11;44(3):1051–67. doi: 10.1093/ije/dyv113 (PMC4521133; doi:10.1093/ije/dyv113)
Supplement: Supplementary Data [file supp_dyv113_suppl_data.zip › ije-2014-04-0428-File004.docx]

**Figure 2** Flow diagram showing the process of identification of published sample size methodology for inclusion in the systematic review

5704 records identified in Web of Science

3027 records identified in PubMed

N=11 excluded

n=1, results in an earlier included paper

n=2, Not applicable in cluster randomised context

n=2, one sample

n=4, Other form of clustering

n=2, methodology already included

8393 records after duplicates removed

72 full-text articles assessed for eligibility

Reference searching

15 identified through reference search on the 61 papers

Reference searching

1 identified through reference search on the 16 papers

Identified ad hoc during the review

N=3 identified

Books, special issue journals, personal collection, experts search

N=4 identified

84 Total papers included in synthesis

77 papers included

61 papers included
