# Supplementary material for: Methods for sample size determination in cluster randomized trials
Source: Int J Epidemiol. 2015 Jul 11;44(3):1051–67. doi: 10.1093/ije/dyv113 (PMC4521133; doi:10.1093/ije/dyv113)
Supplement: Supplementary Data [file supp_dyv113_suppl_data.zip › ije-2014-04-0428-File002.docx]

**Identification and inclusion of papers**

Eligibility

Papers were eligible for inclusion in the review if they described methods for estimating the required sample size, or proposed adaptations to existing approaches, for cluster randomized trials. Designs where clustering occurred, not as a result of randomization but as a consequence of the intervention, in one or both arms of the trial, for example psychological treatments provided in a group setting were excluded. There were no date restrictions on the searches; however, the reports were restricted to the English language due to limited resources for translation.

Data sources

Electronic searches of online databases (PubMed and Web of Science), personal collections of articles on sample size for cluster randomized trials, key text books on cluster randomized trials, searches of special issue journals,^17-20^ and discussions with experts in the field of cluster randomized trials (see acknowledgements) were used. The references of all included papers were searched until no more eligible papers were identified.

Search strategy

Figure 1 describes the search terms used for the electronic databases. Before implementation the search terms were validated using a hand search of all articles published in Statistics in Medicine between January 1982 and March 2011. Relevant hand selected articles were compared against the proposed search terms to ensure they would be identified during the review; the search terms were then refined where necessary.

FIGURE 1 HERE

Data collection and management

A database was created in Microsoft Access 2010 to store and organise the details of all references identified from the electronic databases and was used to manage the results.

A data abstraction form was created to collect information from each eligible paper about: the trial design features; formulae described; the within cluster correlation measure used; the assumptions underpinning the methodology; the simulation procedures used to evaluate the methodology; the strengths and weaknesses as stated by the authors; and any extensions available to the methodology.

All data abstraction was performed by CR; a selection of 10 papers was independently abstracted by SE and AC for quality control.

The search was completed in July 2012 and 84 papers were identified (figure 2) published between 1978 and 2013, with 29 (33%) published in Statistics in Medicine, and 7 (8%) in Biometrics.

FIGURE 2 HERE
